# Ocular diagnostics and occipital neurovascular coupling in ocular hypertension and open angle glaucoma

**DOI:** 10.3389/fnins.2025.1689655

**Published:** 2025-12-12

**Authors:** Dario Messenio, Ester Luconi, Rebecca Re, Patrizia Boracchi, Roberto Colombo, Ester Riva, Lorenzo Spinelli, Davide Contini, Rinaldo Cubeddu, Elia M. Biganzoli, Alessandro Torricelli, Giuseppe Marano

**Affiliations:** 1 Department of Clinical Sciences, Eye Clinic, ASST Fatebenefratelli Sacco Hospital, University of Milan, Milan, Italy; 2 Department of Biomedical Sciences for Health, University of Milan, Milan, Italy; 3 Dipartimento di Fisica, Politecnico di Milano, Milan, Italy; 4 Istituto di Fotonica e Nanotecnologie, Consiglio Nazionale delle Ricerche, Milan, Italy; 5 Medical Statistics Unit, Department of Biomedical and Clinical Sciences, University of Milan, Milan, Italy

**Keywords:** glaucoma, time domain functional near-infrared spectroscopy, hemodynamic response profiling, diagnosis, neurovascular coupling (NVC)

## Abstract

**Introduction:**

The relationship between glaucoma and neurovascular coupling in the visual cortex has yet to be fully explored and understood. This study employs the time-domain (TD) functional near-infrared spectroscopy (fNIRS) technique to noninvasively monitor the hemodynamic response function (HRF) in the visual cortex.

**Methods:**

203 eyes (104 subjects, 46 females, 58 males): 44 with ocular hypertension (OHT), 38 with open-angle glaucoma (OAG), 54 with normal tension glaucoma (NTG), and 67 without abnormal/pathologic condition, were analyzed. All subjects had a complete eye examination, including Goldmann tonometry, computerized visual field optical coherence tomography, pattern electroretinogram, and visual evoked potentials. Visual cortex HRF was assessed by TD-fNIRS using a standard stimulation protocol (reversed checkerboard at 10 Hz). Multivariate statistical analysis was performed to obtain groups (clusters) of eyes based on the respective TD-fNIRS parameters. The relationships between the clusters and the diagnostic groups were assessed by comparing the distributions of the former ones among healthy, hypertensive and glaucomatous eyes.

**Results:**

We found six clusters of eyes, five representing eyes with consistent measurements of HRF amplitudes across acquisition channels (left/right hemisphere) and repeated stimuli, distinguished by distinct magnitudes of neurovascular coupling. The sixth cluster included all the cases of incoherent HRF patterns. Evidence of a different distribution between glaucomatous and healthy eyes was found (*p* = 0.0009), suggesting that high levels of neurovascular coupling are less likely to be observed in NTG and OAG groups.

**Conclusion:**

Occipital TD-fNIRS could be fruitfully implemented in a clinical setting to provide significant and easy-to-get insights on neurovascular dynamics, supporting the differential diagnosis of glaucomatous patients. Our findings highlight the importance of the underappreciated correlates between glaucoma and overall neurologic status.

## 1 Introduction

Open-angle glaucoma (OAG) is a multifactorial optic neuropathy characterized by a progressive loss of retinal ganglion cells (RGC), changes in optic disk morphology, and visual field defects (Weinreb et al., 2016). Intraocular pressure (IOP) is a recognized risk factor for the development and progression of glaucomatous damage (Kass et al., 2002; Medeiros et al., 2008), even though, in some cases, the reduction of IOP is insufficient to slow or halt the progression of the disease. Despite well-controlled IOP, in many patients, the disease progresses, particularly in normal tension glaucoma (NTG), i.e., OAG with IOP inferior to 21 mmHg before hypotonizing therapy (Drance et al., 2001). On the contrary, many subjects with IOP above the average of those with ocular hypertension (OHT) do not develop glaucomatous damage. IOP alone does not explain the pathogenesis of glaucoma, and the etiology of glaucoma itself is still unclear. In glaucoma, characteristic changes of the optic nerve head and the retinal ganglion cells occur, resulting in typical optic disk alterations, recently analyzed by optical coherence tomography (OCT; Kanamori et al., 2003). Similarly, but with a latency of onset of months or years, typical visual field alterations occur (Wua and Medeiros, 2018; Zhang et al., 2017).

As the optic nerve is a portion of white matter, a lesion at its head affects RGC, and this can lead to direct anterograde degeneration of the optic nerve, chiasma, optic tracts up to the geniculate nucleus (Yucel et al., 2001; Yucel et al., 2003), and the visual cortex, as demonstrated by experimental and autoptic human studies (Lawlor et al., 2018; Yücel and Gupta, 2008). So, glaucomatous neurodegeneration has progressively shifted from being considered a purely ocular disorder to being recognized as a disease affecting the entire visual pathway, from the retina to the occipital cortex.

Moreover, the brain is highly vascularized to respond adequately to the metabolic demands due to the neurovascular coupling (NVC). This mechanism links neural brain activation and a corresponding increase in cerebral blood flow (Huneau et al., 2015; Phillips et al., 2016). Various pathological conditions, particularly those disrupting the neurovascular unit, might alter the permanent adaptation of blood supply to local energy needs at the cerebral level (Girouard and Iadecola, 2006). Understanding the interplay between structural, functional, and neurovascular mechanism across the ocular-cortical continuum is essential to clarify glaucoma pathophysiology and to identify novel biomarkers and therapeutic targets.

The NVC can be studied non-invasively using various techniques, such as functional magnetic resonance imaging (fMRI), which relies on blood oxygenation level-dependent (BOLD) signal evaluation (Duncan et al., 2007). It has already been employed to understand the functional cerebral involvement in glaucomatous patients (Wang et al., 2016; Zhang et al., 2012). Subjects with advanced glaucoma can show visual field alteration at the corresponding locations of the flattened cortex (Qing et al., 2010). Recent advances in neuroimaging support this broader perspective. Haykal et al. (2022) demonstrated, using neurite orientation dispersion and density imaging (NODDI), that glaucoma is associated with microstructural degeneration of the pre-geniculate visual pathways, characterized by reduced neurite density and altered orientation dispersion, indicating that glaucomatous damage extends well beyond the retina and optic nerve (Haykal et al., 2022). Similarly, Carvalho et al. (2022) investigated cortical functional plasticity using population receptive field (pRF) mapping with fMRI, showing reduced blood BOLD in glaucomatous patients, together with local shifts and enlargements of pRFs in the early visual cortex. Despite preserved large-scale retinotopy, these findings reveal residual cortical reorganization in adult glaucoma, pointing to adaptive but potentially maladaptive changes in neurovascular demand (Carvalho et al., 2022).

At the network level, Demaria et al. (2021) explored resting-state functional connectivity. They reported preserved global communication across the brain but significant local changes in the lingual gyrus, an occipital hub whose centrality correlated with binocular visual field sensitivity. These results suggest that while global brain connectivity remains intact, local network alterations within the visual cortex reflect disease severity and may shape regional neurovascular responses (Demaria et al., 2021).

While fMRI is a valuable tool in comprehending glaucoma involvement at the cerebral level, it is an expensive exam with a long acquisition time. The interpretation and analysis of fMRI images and signals need specialized personnel. Moreover, it is time-consuming. For all these reasons, fMRI is not widely employed for glaucoma applications.

Functional near-infrared spectroscopy (fNIRS) is a non-invasive optical technique for monitoring NVC in humans, that has some advantages over fMRI: the instrument has limited dimensions and costs, and can be placed directly at the point of care. Also, interpretating the fNIRS signal is much faster and easier once a routine is established and provided to the clinician (Obrig and Villringer, 2003; Ferrari and Quaresima, 2012; Jacques, 2013; Hoshi, 2016; Torricelli et al., 2014a,b; Koo et al., 2015; Kashikura et al., 2001). In particular, with the time-domain (TD) approach, it is possible to retrieve the absolute values of the concentration of oxy-hemoglobin (O_2_Hb) and deoxy-hemoglobin (HHb; Ferrari and Quaresima, 2012; Jacques, 2013; Hoshi, 2016; Torricelli et al., 2014b) related to the only cerebral cortex. These hemodynamic parameters are the expression of the NVC. Indeed, during the TD-fNIRS evaluation, the subject is shown appropriate visual stimulation. At the visual cortical level, the typical NVC response (activation) is manifested with an increase in O_2_Hb with a contextual and less pronounced decrease in HHb. This behavior is modeled with the so-called hemodynamic response function (HRF). The typical visual stimulation provided is a checkerboard or alternating bars (as is also used during the recording of Visual Evoked Potentials, VEPs; Hoshi, 2016; Torricelli et al., 2014a,b; Koo et al., 2015). VEPs are electrophysiological signals evoked by visual stimuli that can be extracted from an electroencephalographic activity from the visual cortex (Kashikura et al., 2001; Wan et al., 2006; Mayhew et al., 2010). There is a linear correlation among VEP amplitudes and a negative correlation of VEP amplitudes with VEP latencies and hemodynamic variations evaluated indirectly by the fNIRS technique (Rovati et al., 2007; Si et al., 2016; Obrig et al., 2002). Moreover, VEP parameters varied with stimulus contrast level (Si et al., 2016).

The relationship between glaucoma and HRF in the visual cortex has yet to be fully explored and understood. In a preliminary study with a relatively small number of glaucoma patients and sleep apnea subjects, the hemodynamic response of the visual cortex, assessed by fNIRS, showed a lower change in O_2_Hb concentration in eight glaucoma and six snores subjects compared to controls (Ward et al., 2018). A recent study found a reduction in TD-fNIRS response in glaucomatous subjects compared to a healthy control group of similar age (Re et al., 2021). Among all tests and exams available for diagnosing glaucoma, ocular electrophysiological tests such as Pattern Electroretinogram (PERG) and VEPs have the advantage of being objective. PERG directly indicates RGC function: it reflects ganglion diffuse rather than focal damage (Re et al., 2021; Bach, 2001; Bach and Hoffmann, 2008; Van den Berg et al., 1986; Bach et al., 1997; Ventura et al., 2005; Banitt et al., 2013; Messenio et al., 2016a; Messenio et al., 2016b; Unsoeld et al., 2001; Bach et al., 2006; Salgarello et al., 1999; Ventura and Porciatti, 2005; Ventura et al., 2012). VEPs characterize the state of the whole visual pathway from the eye to the visual cortex (Odom et al., 2016). Subjects with a suspicion of NTG but with quite normal visual field had P100 amplitude VEPs reduced, P100 latency slightly delayed, as well as a slight reduction in the amplitude of the PERG (Parisi et al., 2001; Karaskiewicz et al., 2014). After 1 year, patients enrolled in the study experienced a limited reduction in IOP; however, the latency and amplitude of the P100 wave VEPs did not show significant changes. This lack of change is understandable, as topical therapy is unlikely to influence the visual pathways’ functionality (strictly neurological). On the contrary, P50 N95 complex amplitudes slightly increased (Parisi et al., 2001; Karaskiewicz et al., 2014).

Neuroimaging and electrophysiological studies have shown that glaucomatous pathology cannot be considered strictly confined to the eye, but a definite post-retinal involvement up to the occipital cortex must be considered. The findings, although still incomplete and to be confirmed, would seem to place this pathology in the context of neurodegenerative diseases, and it is helpful to analyze it from this perspective. Based on this background, the ultimate research goal was to address a specific scientific question: are ocular hypertension and open-angle glaucoma associated with measurable alterations in neurovascular coupling along the ocular-to-visual pathway? Our testable hypothesis was that patients with ocular hypertension and glaucoma would exhibit impairments in neurovascular coupling parameters, which would correlate with both functional visual field changes and structural alterations detected by ocular imaging.

In this study, the primary goals were to provide valuable insights into the functional response of the visual cortex in OHT, NTG/OAG subjects, and evaluate the consistency of measurements across repeated stimuli and recording channels, to check the stability and reproducibility of the measurements. To this end, 203 eyes with distinct conditions, i.e., healthy, OHT, NTG, and OAG; were exposed to visual stimulation, and the HRF was evaluated using the TD-fNIRS technique. To deal with the high dimensionality of these recordings, multivariate statistical methods were adopted for data analysis. In fact, these methods allow summarizing information from large variable sets, while requiring few assumptions about the HRF (Gemignani et al., 2018). Accordingly, they were used to classify the 203 eyes into groups (clusters) based on the patterns emerging from the HRF analysis. Then, the relationship between clusters, the pathological condition and the glaucoma staging system two (GSS2) were evaluated. These steps provided preliminary results for assessing the diagnostic accuracy of imaging measurements in the following studies. Finally, as a secondary goal, we performed an exploratory inspection of the correlation between the consistent patterns emerging from the primary analysis and the morphometric, functional, and electro-functional ocular parameters.

## 2 Materials and methods

### 2.1 Ethical statement

The study was approved by the Sacco Hospital Medical Ethical Committee (n. 0018034, 07/07/2015) and by the Ministry of Health (DGDMF. VI/P/I.5.m.i.2/2015/1022), and was conducted in compliance with the Declaration of Helsinki. All subjects gave their written informed consent to participate in the study after being informed of its nature and possible implications.

### 2.2 Subjects

The observational study included participants with ocular hypertension, normal tension glaucoma, and hypertensive glaucoma, as well as healthy controls. The inclusion and exclusion criteria were adopted from those reported by Re et al. (2021). Eligible subjects were adults with best-corrected visual acuity ≥ 0.8, normal anterior and posterior segment findings, and intraocular pressure (IOP) < 21 mmHg in both eyes. Only eyes with reliable visual fields and, in the normal group, normal optic disk morphology, were included. Exclusion criteria included any ocular pathology (other than ocular hypertension or open-angle glaucoma in the study groups), prior ocular surgery or trauma, systemic vascular or neurological disorders, diabetes, uncontrolled hypertension, and use of topical or systemic agents influencing IOP or ocular blood flow. Refractive errors > +/− 3 D spherical equivalent or > 1.5 D astigmatism were also excluded. All participants underwent a complete ophthalmological examination before enrollment. The eyes of the eligible subjects were classified as ocular hypertensive (*N* = 44), OAG (*N* = 38), NTG (*N* = 54), and healthy (*N* = 67 eyes). The perimetric stadiation was expressed by a score from 0 to 5.

### 2.3 Visual field data acquisition

Octopus 101: G2 program for glaucoma (Interzeag, Schlieren, Switzerland) was performed: GSS2 perimetric staging was used, which considers visual field defects by analysing perimetric indices: mean defect (MD) and corrected loss variance (CLV). Perimeter staging was carried out according to the Brusini Glaucoma Staging System classification: the GSS 2 classifies visual field defects into three types: generalized, localized, and mixed, and five levels of severity: 1 = mild to 5 = severe (Brusini and Filacorda, 2006). The stages were considered without analysing the subcategories (localized, mixed, and generalized defects).

### 2.4 Electrophysiological data acquisition

PERGs were recorded simultaneously with VEPs, with 30-min and 15-min black-and-white checkerboard pattern stimulus, 45 cd/m2 mean luminance, reversing two times per second (square wave reversal) or counter-phased at 1 Hz (thus evoking transient responses) at 98% contrast between black and white squares. Signals were amplified (50,000 times) and filtered (pass band 1–100 Hz). The stimuli were generated on a cathode-ray tube monitor subtending 24° at a viewing distance of 114 cm (Pazos et al., 2017). Parameters were recorded using the Biomedica Mangoni SNC (Pisa, Italy).

The following parameters were considered (in the 30- and 15-min arc stimulation): VEPs: amplitude and latency of the P100 wave; PERG: amplitude and latency of the P50-N95 complex.

### 2.5 OCT data acquisition

Peripapillary Retinal Nerve Fiber Layer (pOCT) and macular ganglion fibers layers (mOCT) imaging was performed using Spectral Domain Optical Coherence Tomography examination (Spectralis OCT Heidelberg Engineering Inc.; Karaskiewicz et al., 2014) analyzing upper (s), nasal (n), lower (i), and temporal (t) pOCT, and lower (i), upper (s), and total (tot) mOCT.

### 2.6 TD-fNIRS data acquisition and analysis

TD-fNIRS acquisitions were accomplished by a multi-channel device developed at the Department of Physics at Politecnico di Milano and approved by the Italian Ministry of Health (Re et al., 2013). According to the 10/20 EEG positioning system, we placed one injection fiber in the OZ position and two detection fibers in the O1 and O2 positions, respectively, using a modified EEG cap and a 3D-printed custom probe (Amendola et al., 2021). The protocol is fully described elsewhere (Re et al., 2021) and, in summary, consists of five repeated visual stimulation cycles (pattern reversal checkerboard, 10 s rest, 10 s visual stimulus, 10 s recovery), performed one eye at a time.

The cortical O_2_Hb and HHb concentrations throughout the whole experiment were calculated as described in Zucchelli et al. (2013) and fitted with a canonical HRF (Uga et al., 2014). From the previous procedure, it was possible to determine, for each eye, each hemisphere, each stimulation, and hemodynamic parameter, the amplitude of the response (A) and the relative delay from the stimulus onset (*τ*). As determined by the experimental protocol, for each eye, the recorded parameters consist of 20 amplitudes and 20 delays, according to signal type (O_2_Hb, HHb), acquisition channel (left/right hemisphere), and repetition of stimulation (1 to 5).

### 2.7 Data analysis

The main goal was to identify the patterns in O_2_Hb and HHb signals through the analysis of the TD-fNIRS parameters A and *τ* (previous paragraph) and to evaluate the association of these patterns with the eye’s condition (OHT, NTG, OAG, and healthy) and GSS2 perimetric staging. To this end, multivariate analysis methods were adopted (Lebart et al., 1995).

In the first step, Multiple Correspondence Analysis (MCA) was adopted. TD-fNIRS parameters were classified into five ordinal classes (quintiles), reported in Supplementary Table 1. Feature selection was performed to improve the efficiency of the analysis. To this end, variables with trivial relationships with the others were excluded using the squared correlation index. Then, MCA was performed. The number of relevant factorial axes was chosen using the scree-plot method, with the index of inertia proposed by Benzecri as a measure of the proportion of total data variability explained by the axes (Greenacre, 2017). Coordinates of eyes and TD-fNIRS parameters on the principal axes were graphically represented by MCA biplots.

Cluster analysis was performed on the coordinates of the eyes on the principal axes. The clustering procedure was hierarchical agglomerative clustering, with Euclidean distance as the dissimilarity measure and the Ward’s minimum-variance method (Ward linkage) as the aggregation criterion (Lebart et al., 1995). The optimal number of clusters was determined by the inertia gain criterion, which identifies the partition beyond which additional clusters result in only marginal increases in explained inertia (Le et al., 2008). A first interpretation of the clusters was performed by evaluating their relative positions within the MCA biplots.

To assess the association between clusters and eye condition, the prevalence of clusters within OHT, NTG, OAG, and healthy eyes was assessed and compared across groups. To this end, a multinomial regression model for clustered data (Touloumis, 2015) was used to account for fellow eyes. All the analyses were performed using the software R release 4.1.2 (R Core Team, 2021) with the packages FactoMineR (Le et al., 2008) and multgee (Touloumis, 2015) added, and KNIME Analytic Platform version 4.5.0 (Berthold et al., 2008).

## 3 Results

The analysis was performed on data from 203 eyes (99 Left, 104 Right) from 104 patients aged from 38 to 84 years (mean: 69.5 years; SD: 9.2 years), of which 46 (44.2%) were females. The results of ophthalmological examinations are summarized in Table 1 and in Supplementary Table 2. Five eyes were excluded due to invalid fNIRS recordings. Among the 99 subjects with recordings available for both eyes, 91 had eyes with the same condition (31 healthy, 22 OHT, 18 OAG, and 25 NTG), and 8 had distinct conditions in the left and right eyes. According to the acquisition protocol, 20 pairs of TD-fNIRS parameters—i.e., response amplitude (A) and delay from stimulus onset (*τ*)—have been recorded for each eye, conditional to two hemoglobin components (O_2_Hb and HHb), two acquisition channels (left/right hemisphere), and five stimuli. The median values of these parameters were reported in Supplementary Table 3 for descriptive purposes.

**Table 1:** Summary of clinical characteristics of 203 eyes in the present study.

	Eye	Clinical classification			
		NORM (*n* = 67)	OHT (*n* = 44)	NTG (*n* = 54)	OAG (*n* = 38)
IOP (mmHg)	L	14.8, 2.1	17.8, 3.2	12.9, 2.6	13.7, 2.9
	R	15.0, 2.1	17.4, 2.8	12.8, 2.8	14.3, 3.5
PAC (μm)	L	550.0, 29.6	581.9, 34.5	537.1, 30.0	559.5, 45.4
	R	549.8, 35.9	578.8, 34.2	537.0, 27.6	548.7, 45.0
MD (dB)	L	2.3 (2.0–3.3)	3.1 (2.4–3.6)	9.3 (5.2–13.5)	9.8 (5.5–18.3)
	R	2.3 (1.7–3.3)	3.2 (2.6–3.9)	8.7 (6.5–10.8)	7.7 (5.9–11.9)
CLV (dB^2^)	L	1.9 (1.4–2.2)	1.6 (1.3–2.0)	5.5 (3.4–7.7)	5.6 (2.9–7.0)
	R	1.8 (1.4–2.1)	1.7 (1.3–2.0)	5.4 (4.3–7.7)	6.3 (3.7–7.5)
mOCT.t (μm)	L	30.0 (29.0–32.5)	31.0 (29.0–33.0)	24.0 (21.0–27.0)	23.5 (20.0–29.0)
	R	30.5 (28.7–33.0)	31.0 (29.0–32.0)	25.5 (24.0–27.0)	25.0 (21.0–28.0)
lVEPs30 (ms)	L	113.0 (108.0–117.0)	114.0 (109.0–117.0)	119.0 (113.0–132.5)	125.5 (119.2–142.2)
	R	110.5 (106.0–115.8)	112.0 (108.0–115.0)	111.0 (107.5–122.5)	119.0 (111.5–120.0)
lVEPs15 (ms)	L	119.0 (111.0–120.0)	118.0 (114.0–120.0)	123.0 (118.0–135.0)	131.5 (124.2–141.0)
	R	118.5 (114.0–120.0)	117.0 (112.0–120.0)	119.0 (117.0–130.0)	120.5 (117.2–127.5)
aVEPS30 (μV)	L	8.4 (6.2–11.7)	8.1 (4.9–10.0)	6.6 (4.6–9.0)	5.3 (3.2–7.1)
	R	10.2 (6.9–13.1)	8.2 (6.3–12.7)	9.1 (5.4–12.6)	7.0 (5.5–8.4)
aVEPs15 (μV)	L	10.1 (7.7–14.2)	7.9 (6.9–12.2)	5.8 (4.4–8.2)	4.4 (2.6–7.7)
	R	12.1 (7.7–13.4)	9.4 (7.3–14.7)	9.5 (5.3–10.6)	5.9 (4.4–8.4)
lPERG30 (ms)	L	58.0 (51.5–62.0)	58.0 (55.0–61.0)	65.0 (56.5–71.0)	56.5 (49.0–70.8)
	R	58.0 (54.0–60.0)	58.0 (55.0–61.0)	60.0 (57.0–65.0)	61.5 (56.5–64.5)
lPERG15 (ms)	L	63.0 (56.0–70.0)	61.0 (57.0–64.0)	62.0 (55.0–73.0)	62.5 (57.0–68.2)
	R	59.0 (56.5–62.0)	61.0 (53.5–65.5)	63.0 (56.0–70.5)	66.0 (59.2–75.8)
aPERG30 (μV)	L	2.3 (1.9–3.0)	3.1 (2.1–3.7)	2.1 (1.6–2.7)	2.1 (1.6–2.4)
	R	2.8 (2.6–3.6)	3.0 (2.2–3.6)	2.5 (1.5–3.2)	2.4 (1.5–2.7)
aPERG15 (μV)	L	2.1 (1.8–2.8)	2.4 (2.0–3.1)	1.7 (1.4–2.3)	1.9 (1.4–2.4)
	R	2.3 (1.9–2.7)	2.3 (2.0–3.1)	1.9 (1.6–2.8)	2.2 (1.4–2.5)

IOP and PAC were summarized using mean and standard deviation. The remainder variables were summarized using median, first and third quartiles (25-th and 75-th centiles, respectively). IOP, inter ocular pressure; PAC, pachimetry; MD, mean defect; CLV, corrected loss variance; mOCT, macular OCT; lVEPs, VEPs latency; aVEPs, VEPs amplitude; lPERG, PERG latency; aPERG, PERG amplitude; L, left eye; R, right eye; NORM, normal; OHT, ocular hypertensive; NTG, normal tension glaucoma; OAG, open angle glaucoma.

### 3.1 Neurovascular patterns

**Figure 1:**
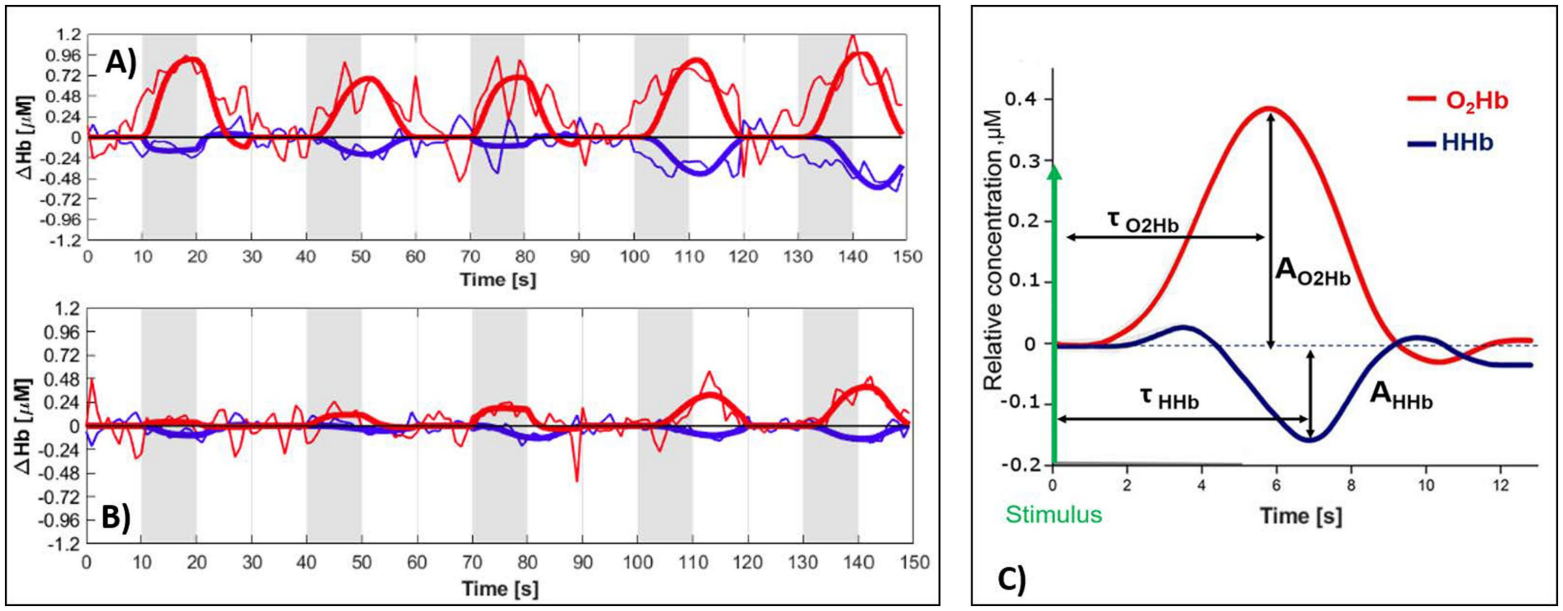
TD-fNIRS time courses (thin line) for O2Hb (red) and HHb (blue) concentration changes. With thick lines: fitting with the HRF model. **(A)** NORM; **(B)** OAG. Stimulation of the right eye, answer of the right hemisphere. The stimulation periods are represented in gray. **(C)** Graphical representation of amplitudes (A_O2Hb_, A_HHb_) and delays (τ_O2Hb_, τ_HHb_) of the HRF for each stimulation.

In Figure 1, with thin lines, an example is presented of the typical TD-fNIRS time course during the five visual stimuli (gray area), i.e., O_2_Hb (red) and HHb (blue), for a NORM eye (A) and an OAG eye (B). The thick lines result from the fit procedure with the HRF model. The NORM group shows a typical increase in O_2_Hb and a decrease in HHb, whereas in OAG this behavior is not replicated. Figure 1C presents a graphical representation of the parameters A and *τ* extrapolated for each hemodynamic parameter and each stimulus given.

**Figure 2:**
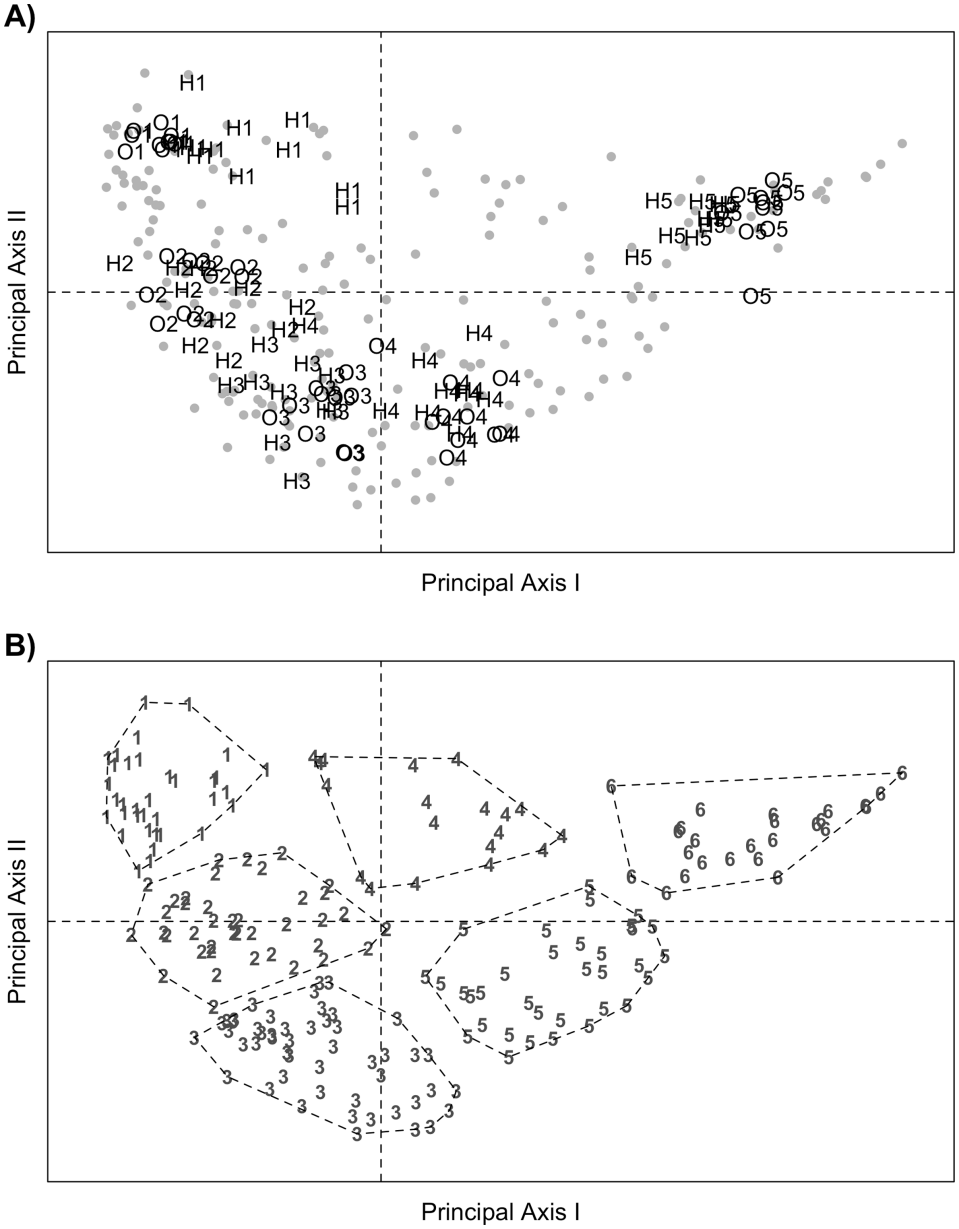
MCA biplots showing the relationships among TD-fNIRS amplitudes. Panel **A**: Eyes are represented by gray points; fNIRS amplitudes, classified in five classes according to their magnitude, are represented by the labels: O1 to O5 (O2Hb component) and H1 to H5 (HHb component). The label O1 represent the values closest to 0 μM; the following labels (O2-O5) represent values gradually more distant from 0 μM. The same holds for the labels H1 to H5. Panel **B**: Eyes are represented with numbers from 1 to 6, according to the pertinent cluster membership. The points belonging to the same clusters are delimited by dashed polygons, so that to provide a clear visualization of the clusters in the figure.

To perform MCA, TD-fNIRS parameters were classified into five ordinal classes. In the feature selection step, all the parameters with a squared correlation index below 0.3 for the first principal axis and below 0.2 for the second principal axis were excluded (Supplementary Figure S1). Therefore, only response amplitudes were included in the subsequent analysis. According to Benzecri’s rule, 75.6% of the total data variability is explained by the first two principal axes, while higher-order axes do not contribute substantially. Therefore, the first two axes were used for producing MCA biplots (Figure 2).

From now on, the ordinal classes of the amplitude parameter will be indicated by O1 to O5 for the O_2_Hb response and H1-H5 for the HHb response. In Figure 2A, it may be noted that the labels O1 and H1 are grouped closely, and the same holds for the following: O2 and H2, O3 and H3, O4 and H4, and O5 and H5. Furthermore, all the labels are sorted in increasing order from the left side of the Figure (O1, H1) to the right (O5, H5). These features suggest the following patterns:

(1) TD-fNIRS amplitudes tend to show similar magnitude across distinct hemispheres and stimuli;
(2) amplitudes of the O_2_Hb component close to (distant from) 0 μM are likely to be observed simultaneously with HHb amplitudes close to (distant from) 0 μM. In other terms, a non-trivial correlation between O_2_Hb and HHb amplitudes may be expected.

In the subsequent step, 6 clusters were identified, including 35, 40, 50, 19, 34, and 25 eyes, respectively. Figure 2B provides valuable support for interpretation. In the figure, clusters 1, 2, 3, 5, and 6 are sorted from left to right, and their position is close to the ordinal classes of TD-fNIRS amplitudes (O1 to O5, H1 to H5). This suggests that, for those clusters, TD-fNIRS amplitudes increase. On the contrary, cluster 4 (top of the figure) is approximately equidistant from all ordinal classes, suggesting that it may represent eyes with mixed amplitude values.

To check the above interpretation, boxplots showing the distribution of TD-fNIRS amplitudes within each cluster were reported: see Supplementary Figure S2. It may be seen that for cluster 1, the boxplots are centered upon the value of 0 μM, while, in clusters 2, 3, 5 and 6 the boxplots show an increasing and a decreasing trend, respectively for the O_2_Hb and HHb components. A further check using partitioning tree methods was performed; see Supplementary Figure S3 and Supplementary Table 4 for more details.

In conclusion, based on the interpretation of the patterns shown in Figure 2 and subsequent data checks, clusters 1, 2, 3, 5, and 6 represent an increasing NVC, with consistent measurements across acquisition channels and stimulus repetition. Instead, cluster 4 represents eyes with incoherent HRFs.

The results presented in this section do not provide a quantitative assessment of the overall degree of NVC across clusters (e.g., distinguishing between “low” and “high” NVC). Achieving this goal is not straightforward due to several experimental factors defined in the study protocol, namely multiple measurements across acquisition channels and the inclusion of fellow eyes. These factors introduce methodological complexities when attempting to determine the optimal combination of measurements, potentially reducing the reliability of the resulting estimates. For these reasons, we chose not to provide a quantitative estimate of the overall NVC level for each cluster. This is not a serious drawback because it does not compromise the fulfillment of the study’s objectives.

### 3.2 Relationships between neurovascular patterns and pathological status of the eye

The distributions of cluster membership within healthy, OHT, and glaucomatous eyes are shown in Table 2. In this table, OAG and NTG subgroups are pooled together to prevent unstable results from hypothesis tests. An evident association between eye condition and clusters emerged (global association test: *p* < 0.0001). More in detail, the percentages of the clusters within the glaucoma group were different as compared to the healthy group (*p* = 0.0009), with a lower prevalence of high and highest NVC in the former one (4.3% vs. 28.4% for high NVC, and 4.3% vs. 22.4% for highest NVC) and, therefore, a higher prevalence of low and lowest NVC (28.3% vs. 6.0% for low NVC, and 28.3% vs. 13.4% for lowest NVC). For the sake of completeness, the distributions of clusters within the OAG and NTG groups, and within the GSS2 perimetric stages are shown in Supplementary Table 5.

**Table 2:** Relationships between clinical classification and the clusters.

**CLUSTER**	**Healthy**	**OHT**	**Glaucoma** **(NTG+OAG)**
**1: lowest NVC**	4 ( 6.0%)	5 (11.4%)	26 (28.3%)
**2: low NVC**	9 (13.4%)	5 (11.4%)	26 (28.3%)
**3: intermediate NVC**	15 (22.4%)	13 (29.5%)	22 (23.9%)
**5: high NVC**	19 (28.4%)	11 (25.0%)	4 ( 4.3%)
**6: highest NVC**	15 (22.4%)	6 (13.6%)	4 ( 4.3%)
**4: incoherent HRF**	5 ( 7.5%)	4 ( 9.1%)	10 (10.9%)
Comparison across groups	reference group	*X^2^ = 1.9, df = 5* *P(> X^2^) = 0.86*	*X^2^ = 20.6, df = 5* P(> X^2^) = 0.0009*

The table shows within-group counts and percentages of eyes belonging to different clusters. Comparisons across groups (OHT VS healthy, Glaucoma vs Healthy) are presented in the last row. est, estimate; CI, confidence interval; NC, neurovascular coupling; HRF, hemodynamic response function; OHT, ocular hypertensive.

Finally, the distributions of the clinical characteristics of eyes and VEPs/PERG parameters for each cluster are summarized in Supplementary Table 6 and Supplementary Figure S4. In brief, the median values of MD and CLV showed a decreasing trend from left to right in the figure, indicating a reduction of these parameters with increasing degree of NVC. Cluster 4 is excluded from this conclusion, because it represents subjects with inconsistent HRF recordings, which prevent a reliable assessment of NVC. The median values of peripapillary OCT (pOCT) parameters were higher in clusters 5 and 6 (highest and higher neurovascular coupling) than in clusters 1 and 2 (lowest and lower), especially in the upper and lower quadrants. The median values of VEPs latencies (lVEPs15 and lVEPs30) were higher in eyes with the lowest/low NVC. The highest median VEP amplitudes were in cluster 5; no differences in PERG latencies and amplitudes emerged among different clusters (Table 3).

**Table 3:** Characterization of clusters according to clinical examination results.

	Cluster					
	1: Lowest neurovascular coupling	2: Low neurovascular coupling	3: Intermediate neurovascular coupling	5: High neurovascular coupling	6: Highest neurovascular coupling	4: Incoherent HRF
IOP (mm)	14.3, 2.7	14.8, 3.2	14.5, 3.0	15.6, 2.8	15.6, 3.5	13.8, 3.9
PAC (μm)	559.2, 37.7	552.4, 34.0	551.5, 38.4	558.7, 37.5	551.7, 43.4	547.9, 37.5
MD (dB)	6.9 (3.2–13.2)	5.1 (3.3–9.0)	3.6 (2.4–7.4)	2.8 (1.7–3.8)	3.2 (1.6–3.7)	6.5 (4.0–15.0)
CLV (dB^2^)	3.7 (1.9–6.5)	3.7 (1.8–5.6)	2.3 (1.8–4.7)	1.6 (1.4–2.2)	1.8 (1.4–2.3)	4.4 (1.9–7.8)
M_OCT (μm)						
Lower	27.0 (24.0–30.5)	25.0 (21.0–30.0)	30.0 (25.0–31.0)	27.0 (24.0–30.5)	25.0 (21.0–30.0)	30.0 (25.0–31.0)
Upper	27.0 (23.5–29.0)	26.0 (22.0–29.0)	29.0 (26.0–32.0)	27.0 (23.5–29.0)	26.0 (22.0–29.0)	29.0 (26.0–32.0)
Total	27.0 (23.5–30.0)	26.0 (21.0–29.0)	29.0 (26.0–32.0)	27.0 (23.5–30.0)	26.0 (21.0–29.0)	29.0 (26.0–32.0)
P_OCT (μm)						
Upper	82.0 (62.5–112.5)	91.0 (77.6–112.2)	103.8 (85.2–117.6)	111.8 (102.0–121.9)	105.0 (100.0–115.0)	83.0 (48.5–106.0)
Nasal	58.0 (41.5–67.0)	63.0 (54.5–79.0)	69.0 (60.2–76.8)	76.0 (64.2–82.0)	68.0 (64.0–80.0)	54.0 (43.0–61.0)
Lower	85.0 (63.0–108.0)	81.5 (65.0–118.4)	109.5 (76.2–123.9)	114.5 (105.5–126.8)	114.0 (102.0–132.0)	73.0 (62.5–110.8)
Temporal	62.0 (49.0–70.0)	56.0 (43.2–65.2)	61.0 (52.0–66.0)	66.0 (60.2–69.0)	63.0 (59.0–71.0)	65.0 (51.0–68.5)
lVEP30 (ms)	120.0 (113.0–133.0)	118.5 (110.8–125.8)	113.5 (109.0–117.0)	114.0 (107.2–118.0)	112.0 (105.0–115.0)	109.0 (107.0–119.5)
lVEP15 (ms)	125.0 (117.5–138.5)	120.0 (116.2–128.5)	119.5 (114.2–125.0)	118.0 (115.0–120.8)	118.0 (112.0–120.0)	119.0 (113.0–121.0)
aVEP30 (μV)	7.8 (5.2–9.9)	7.1 (4.6–11.2)	7.8 (5.4–9.9)	8.4 (6.1–14.2)	8.4 (6.8–11.8)	6.5 (5.6–11.4)
aVEP15 (μV)	7.2 (3.9–9.4)	7.0 (4.6–11.3)	8.9 (5.8–11.4)	11.4 (7.3–15.1)	9.4 (7.7–13.5)	8.7 (5.7–11.8)
lPERG30 (ms)	54.0 (50.5–61.5)	58.5 (56.0–63.0)	61.0 (57.0–65.0)	58.0 (55.0–62.0)	58.0 (54.0–61.0)	61.0 (56.0–67.0)
lPERG15 (ms)	63.0 (58.5–68.0)	60.5 (55.0–68.5)	62.0 (55.0–69.8)	62.0 (57.5–67.8)	62.0 (58.0–65.0)	60.0 (55.5–68.0)
aPERG30 (μV)	2.4 (1.9–3.2)	2.4 (1.6–2.8)	2.8 (1.9–3.4)	2.8 (2.0–3.4)	2.8 (2.2–3.5)	2.5 (1.5–3.0)
aPERG15 (μV)	1.9 (1.4–2.4)	2.2 (1.7–2.7)	2.3 (1.9–2.8)	2.1 (1.8–2.8)	1.9 (1.7–2.4)	2.2 (1.6–2.5)

IOP and PAC were summarized using mean and standard deviation, and the remainder variables were summarized by median and quartiles. M_OCT, macular OCT; P_OCT, peri-papillary OCT; lVEPs, VEPs latency; aVEPs, VEPs amplitude; lPERG, PERG latency; aPERG, PERG amplitude; HRF, hemodynamic response function.

## 4 Discussion

Chronic simple glaucoma is a neurodegenerative disease characterized by progressive loss of RGCs resulting in alterations in optic disk morphology and pathognomonic visual field defects. Although glaucoma is commonly associated with an elevation of IOP above 21 mmHg, glaucomatous-type changes can also occur below this value, suggesting that we are dealing with two distinct pathologies with similar, though not overlapping, damage. While lowering IOP remains the gold standard for OAG therapy, it is not the only parameter sufficient to evaluate the clinical stabilization of the disease. Especially in NTG, other systemic variables that determine a clear insufficiency of hypotonizing therapy alone aimed at stabilizing the damage come into play (Medeiros et al., 2008; Drance et al., 2001; Kanamori et al., 2003; Tanna and Desai, 2014).

Moreover, damages at the level of the thalamus’s lateral geniculate nucleus and the primary visual cortex were also found (Yucel et al., 2001; Yucel et al., 2003; Lawlor et al., 2018; Yücel and Gupta, 2008). Thus, other pathogenetic mechanisms are then also hypothesized, and glaucomatous pathology could be inscribed, similarly to Alzheimer’s disease and Parkinson’s disease, in the broad group of neurodegenerative diseases. Therapeutically, the two diseases also differ: in OAG, the gold standard of treatment is to lower IOP pharmacologically and, less frequently, surgically, whereas in NTG, the latter treatment is practically the only one that can slow disease progression (Mallick et al., 2016). Finally, there are subjects with high IOP but no morphological or functional damage to the optic disk: these are the so-called OHT. Most population-based studies have shown that 9.5–17.4% of OHT eyes develop primary open-angle glaucoma without treatment over 5 years (Thomas et al., 2003; Kass et al., 2021). Thus, the mere assessment of IOP is not a reliable index for evaluating the clinical stabilization of the disease.

In the first step of the analysis, TD-fNIRS estimates of peak amplitude showed similar magnitudes across brain regions (left/right hemispheres), signals (O_2_Hb, HHb), and repeated stimuli. These patterns reflect the typical hemodynamic response of the cerebral cortex, i.e., an increase in O_2_Hb with a contextual decrease in HHb. Notably, the consistency of responses to distinct stimuli suggests that TD-fNIRS testing has good repeatability. From the subsequent analysis, we see an association between NIRS clusters and the presence of glaucomatous pathology; there is a higher prevalence of clusters with low occipital NVC within patients with NTG and OAG, as compared to high occipital NVC, confirming the data in the literature that place glaucoma in the neurodegenerative pathologies with involvement of post-retinal structures (in this case the occipital cortex). In contrast, clusters with intermediate or high response prevail in the control group (healthy subjects). In OHT, the distribution in all clusters could be interpreted as indicating the possibility of conversion to glaucoma, but longitudinal studies are needed to confirm this hypothesis.

By analyzing the MD and CLV perimeter indexes and the resulting GSS2 perimeter staging compared to the TD-fNIRS clusters, it can be seen that at stages 0 and 1, i.e., in the presence of a practically normal visual field, the TD-fNIRS responses prevail in clusters 5 and 6, i.e., where the neurovascular coupling is higher. In contrast, in the case of major visual field damage (stages two and upwards), the prevailing TD-fNIRS response occurs in clusters 1, 2, and 3 (minor neurovascular coupling), while little or not at all in clusters 5 and 6. It must be emphasized that the visual field examination is subjective and prone to error, even though the subjects had considerable experience performing the perimetric exam. On the contrary, no significant differences were found when analyzing the TD-fNIRS responses by hemisphere. This could be explained by the pattern of visual pathways (crosses those of nasal origin) and the double cortical representation, which could have masked eventual lateralization of damage. As far as OHT subjects are concerned, they are evenly distributed in all examined NIRS clusters. Longitudinal studies will be needed to discriminate better which of them are likely to develop glaucoma.

Regarding the other variables, OCT values are higher (normal) when there is greater NVC. Less obvious are the results of electrophysiological examinations, although a greater impairment of VEPs (increased latencies) should be noted in presence of low NVC: evidently, the conduction velocity of the visual pathways also decreases as occipital impairment increases. In fact, while it is unlikely that there could be any relationship between retinal ganglion cell function and occipital NVC, it is more likely that the conduction of the visual pathways analyzed by the VEPs may have points of contact with the function of nearby visual areas. However, these results need to be interpreted in the context of further studies.

This work aimed to provide a first clinical indication, based on a sufficiently broad case history, of the presence of a neurological damage component in glaucomatous disease. To achieve this, we employed the TD-fNIRS technique to non-invasively monitor glaucomatous patients’ HRF in the visual cortex. The functional response of the visual cortex was characterized by performing multiple correspondence analysis and cluster analysis on TD-fNIRS parameters (amplitudes and delays). Six eye profiles were identified, of which five represent different amplitudes of NVC, and the sixth one represents incoherent hemodynamic response patterns. In eyes with greater damage, neurovascular coupling is almost reduced. TD-fNIRS has proven to be a new experimental tool that, in addition to an eye examination, could provide meaningful answers from a diagnostic point of view.

### 4.1 Future directions

Future developments of the study aim to expand the patient cohort and to perform a longitudinal assessment of near-infrared spectroscopy (NIRS) as a non-invasive tool to evaluate occipital hemodynamic and oxygenation in patients with hypertension and open angle glaucoma, potentially establishing new functional biomarkers for early diagnosis and monitoring of disease progression.

## Data Availability

The data analyzed in this study is subject to the following licenses/restrictions: the data supporting the findings of this study are available from the corresponding author upon reasonable request and subject to institutional approval and signature of a data transfer agreement. Summary data corresponding to the tables and figures in the manuscript are available at on Zenodo at 10.5281/zenodo.17630012 under CC-BY license. The R code used to perform the statistical analyses is available without undue restriction. Requests to access these datasets should be directed to dario.messenio@virgilio.it.

## Supplementary material

SUPPLEMENTARY FIGURE S1 Feature selection on TD-FNIRS parameters. Peak amplitudes are represented with black dots, peak delays with gray diamonds. The X-axis and the Y-axis correspond to the squared correlation of the above parameters with the first and the second principal axes, respectively. Values close to 0 indicate little importance of the variable, while values close to 1 indicate a greater one. Therefore, the less representative variables are those in the lower left part of the figure.

SUPPLEMENTARY FIGURE S2 Distribution of TD-fNIRS amplitudes within clusters. The boxplots are arranged in four panels, according to hemoglobin component (O2Hb and HHb, respectively in **A, B** and **C, D**) and acquisition channel (left and right hemispheres, respectively in panels **A, C** and **B, D**. Within each panel, boxplots show are arranged according to 6 clusters, and 5 repetition of stimuli.

SUPPLEMENTARY FIGURE S3 Tree diagram representing the partitioning of the sample based on TD-fNIRS peak amplitudes. A partition tree is a hierarchical structure representing a set of subsequent partitions performed to the statistical units within a sample (eyes in the current case). In the tree diagram represented here, the node at the top represents the whole sample of 203 eyes. The following nodes represent subsets of the whole sample obtained by applying partitioning rules. For example, the 203 eyes in the top node were partitioned in two subsets: those with peak amplitude observed after the 4th stimulation in the 2nd channel, i.e. right hemisphere (AMP_4_2) equal to 1, 2 or 3 (left side) and those with AMP4_2_OHB equal to 4 and 5 (right side). The circles at the bottom of the figure are called leaves or terminal nodes. Here, 8 terminal nodes are placed on the bottom of the tree, including 32, 12, 32, 47, 12, 9, 27, and 32 eyes, respectively. The number reported within each leaf is the cluster with the highest frequency within the respective subset of eyes: cluster 1, 2, 2, 3, 3, 5, 5, 6, respectively. The proportions reported below the terminal nodes are the classification errors. By considering this index, it may be seen that the majority of the eyes assigned to the first terminal node (27/32) belong to cluster 1 (lowest neurovascular coupling), while for the second and third terminal nodes, eyes belonging to cluster 2 (low neurovascular coupling) are prevalent over the other ones (5/12 and 18/32, respectively).

SUPPLEMENTARY FIGUREs S4 Boxplots showing the distribution of MD and CLV Perimetric Indices for each cluster. MD, mean defect; CLV, corrected loss variance; M_OCT, macular OCT; P_OCT, peripapillary OCT; lVEPs, VEPs latency; aVEPs, VEPs amplitude; lPERG, PERG latency; aPERG, PERG amplitude.

SUPPLEMENTARY FIGUREs S5 Boxplots showing the distribution of MD and CLV Perimetric Indices for each cluster. MD, mean defect; CLV, corrected loss variance; M_OCT, macular OCT; P_OCT, peripapillary OCT; lVEPs, VEPs latency; aVEPs, VEPs amplitude; lPERG, PERG latency; aPERG, PERG amplitude.

SUPPLEMENTARY FIGUREs S6 Boxplots showing the distribution of MD and CLV Perimetric Indices for each cluster. MD, mean defect; CLV, corrected loss variance; M_OCT, macular OCT; P_OCT, peripapillary OCT; lVEPs, VEPs latency; aVEPs, VEPs amplitude; lPERG, PERG latency; aPERG, PERG amplitude.
