# Supplementary material for: Ocular diagnostics and occipital neurovascular coupling in ocular hypertension and open angle glaucoma
Source: Front Neurosci. 2025 Dec 12;19:1689655. doi: 10.3389/fnins.2025.1689655 (PMC12740935; doi:10.3389/fnins.2025.1689655)
Supplement: Supplementary file 7 [file Table_1.docx]

**Supplementary Table ST1. Definition of the classes of TD-fNIRS parameters**

The classes were defined for each recorded parameter, A and τ, for each combination of: signal type (O_2_Hb, HHb), acquisition channel (left/right hemisphere), and repetition of stimulation (1 to 5). O_2_HB : oxy-hemoglobin; HHb: deoxy-hemoglobin.

|  | **CLASS** | | | | |  |
| --- | --- | --- | --- | --- | --- | --- |
|  | **1** | **2** | **3** | **4** | **5** |  |
| **O_2_HB**  **LEFT HEMISPHERE**  stimulation 1  stimulation 2  stimulation 3  stimulation 4  stimulation 5  **RIGHT HEMISPHERE**  stimulation1  stimulation 2  stimulation 3  stimulation 4  stimulation 5 | A < 0.1390 µM  A < 0.1349 µM  A < 0.1444 µM  A < 0.0818 µM  A < 0.1324 µM  A < 0.0992 µM  A < 0.1390 µM  A < 0.1750 µM  A < 0.1744 µM  A < 0.2346 µM | 0.1390 µM < A < 0.2965 µM  0.1349 µM < A < 0.3188 µM  0.1444 µM < A < 0.3762 µM  0.0818 µM < A < 0.3598 µM  0.1324 µM < A < 0.3872 µM    0.0992 µM < A < 0.3868 µM  0.1390 µM < A < 0.4120 µM  0.1750 µM < A < 0.3902 µM  0.1744 µM < A < 0.3704 µM  0.2346 µM < A < 0.4490 µM | 0.2965 µM < A < 0.5910 µM  0.3188 µM < A < 0.6296 µM  0.3762 µM < A < 0.5970 µM  0.3598 µM < A < 0.6042 µM  0.3872 µM < A < 0.6088 µM  0.3868 µM < A < 0.6088 µM  0.4120 µM < A < 0.6544 µM  0.3902 µM < A < 0.5976 µM  0.3704 µM < A < 0.6124 µM  0.4490 µM < A < 0.7246 µM | 0.5910 µM < A < 0.8970 µM  0.6296 µM < A < 0.8536 µM  0.5970 µM < A < 0.9268 µM  0.6042 µM < A < 0.8574 µM  0.6088 µM < A < 0.9062 µM  0.6088 µM < A < 0.9422 µM  0.6544 µM < A < 1.0344 µM  0.5976 µM < A < 0.9380 µM  0.6124 µM < A < 0.9944 µM  0.7246 µM < A < 0.9292 µM | A > 0.8970 µM  A > 0.8536 µM  A > 0.9208 µM  A > 0.8574 µM  A > 0.9422 µM  A > 0.9422 µM  A > 1.0344 µM  A > 0.9380 µM  A > 0.9944 µM  A > 0.9292 µM |  |
| **HHb**  **LEFT HEMISPHERE**  stimulation 1  stimulation 2  stimulation 3  stimulation 4  stimulation 5  **RIGHT HEMISPHERE**  stimulation 1  stimulation 2  stimulation 3  stimulation 4  stimulation 5 | A > 0.0268 µM  A > -0.0180 µM  A > -0.0494 µM  A > 0.0024 µM  A > 0.0000 µM  A > -0.0102 µM  A > -0.0154 µM  A > -0.0628 µM  A > -0.0228 µM  A > -0.0454 µM | -0.0916 µM < A < 0.0268 μM  -0.1348 µM < A <-0.0180 μM  -0.1436 µM < A <-0.0494 μM  -0.0986 µM < A < 0.0024 μM  -0.1096 µM < A < 0.0000 μM  -0.1027 µM < A <-0.0102 µM  -0.1152 µM < A <-0.0154 µM  -0.1528 µM < A <-0.0628 µM  -0.1320 µM < A <-0.0228 µM  -0.1498 µM < A <-0.0454 µM | -0.1704 µM < A <-0.0916 µM  -0.2062 µM < A <-0.1348 µM  -0.2413 µM < A <-0.1436 µM  -0.1802 µM < A <-0.0986 µM  -0.2142 µM < A <-0.1096 µM  -0.1740 µM < A <-0.1027 µM  -0.2074 µM < A <-0.1152 µM  -0.2224 µM < A <-0.1528 µM  -0.2294 µM < A <-0.1320 µM  -0.2388 µM < A <-0.1498 µM | -0.3116 µM < A <-0.1704 µM  -0.3632 µM < A <-0.2062 µM  -0.3873 µM < A <-0.2413 µM  -0.3472 µM < A <-0.1802 µM  -0.3762 µM < A <-0.2142 µM  -0.3002 µM < A <-0.1740 µM  -0.3134 µM < A <-0.2074 µM  -0.3476 µM < A <-0.2224 µM  -0.3878 µM < A <-0.2294 µM  -0.3924 µM < A <-0.2388 µM | A <-0.3116 µM  A <-0.3632 µM  A <-0.3873 µM  A <-0.3472 µM  A <-0.3762 µM  A <-0.3002 µM  A <-0.3134 µM  A <-0.3476 µM  A <-0.3878 µM  A <-0.3924 µM |  |

|  | **CLASS** | | | | |
| --- | --- | --- | --- | --- | --- |
|  | **1** | **2** | **3** | **4** | **5** |
| **O_2_HB**  **LEFT HEMISPHERE**  stimulation 1  stimulation 2  stimulation 3  stimulation 4  stimulation 5  **RIGHT HEMISPHERE**  stimulation 1  stimulation 2  stimulation 3  stimulation 4  stimulation 5 | τ < 1.4834 s  τ < 1.7664 s  τ < 1.2990 s  τ < 1.8373 s  τ < 2.4068 s  τ < 1.8532 s  τ < 2.7432 s  τ < 1.5034 s  τ < 2.0312 s  τ < 2.7990 s | 1.4834 s < τ < 3.0662 s  1.7664 s < τ < 3.6484 s  1.2990 s < τ < 3.6456 s  1.8373 s < τ < 3.9170 s  2.4068 s < τ < 4.4298 s    1.8532 s < τ < 3.6964 s  2.7432 s < τ < 4.6156 s  1.5034 s < τ < 3.7672 s  2.0312 s < τ < 3.7920 s  2.7990 s < τ < 5.6586 s | 3.0662 s < τ < 4.8468 s  3.6484 s < τ < 5.0622 s  3.6456 s < τ < 5.2384 s  3.9170 s < τ < 5.3798 s  4.4298 s < τ < 5.9404 s  3.6964 s < τ < 5.3676 s  4.6156 s < τ < 5.9298 s  3.7672 s < τ < 5.2834 s  3.7920 s < τ < 5.4604 s  5.6586 s < τ < 5.6586 s | 4.8468 s < τ < 6.5268 s  5.0622s < τ < 7.0732 s  5.2384 s < τ < 7.3860 s  5.3798 s < τ < 7.1384 s  5.9404 s < τ < 7.5560 s  5.3676 s < τ < 7.0246 s  5.9298 s < τ < 7.6502 s  5.2834 s < τ < 6.6372 s  5.4604 s < τ < 7.0592 s  5.6586 s < τ < 7.1708 s | τ > 6.5268 s  τ > 7.0732 s  τ > 7.3860 s  τ > 7.1384 s  τ > 7.5560 s  τ > 7.0246 s  τ > 7.6502 s  τ > 6.6372 s  τ > 7.0592 s  τ > 7.1708 s |
| **HHb**  **LEFT HEMISPHERE**  stimulation 1  stimulation 2  stimulation 3  stimulation 4  stimulation 5  **RIGHT HEMISPHERE**  stimulation 1  stimulation 2  stimulation 3  stimulation 4  stimulation 5 | τ < 1.3404 s  τ < 1.7976 s  τ < 1.9261 s  τ < 2.0046 s  τ < 1.5968 s  τ < 0.5810 s  τ < 1.3240 s  τ < 1.9168 s  τ < 2.1188 s  τ < 1.8308 s | 1.3404 s < τ < 3.4520 s  1.7976 s < τ < 4.0588 s  1.9261 s < τ < 4.7552 s  2.0046 s < τ < 4.3888 s  1.5968 s < τ < 4.4270 s    0.5810 s < τ < 3.2350 s  1.3240 s < τ < 4.4202 s  1.9168 s < τ < 4.4433 s  2.1188 s < τ < 4.7356 s  1.8308 s < τ < 4.5492 s | 3.4520 s < τ < 5.4916 s  4.0588 s < τ < 6.1077 s  4.7552 s < τ < 6.3938 s  4.3888 s < τ < 6.0480 s  4.4270 s < τ < 5.9516 s  3.2350 s < τ < 5.8472 s  4.4202 s < τ < 6.2332 s  4.4433 s < τ < 6.0960 s  4.7356 s < τ < 6.5580 s  4.5492 s < τ < 6.3714 s | 5.4916 s < τ < 8.2008 s  6.1077 s < τ < 7.8176 s  6.3938 s < τ < 7.8952 s  6.0480 s < τ < 8.4772 s  5.9516 s < τ < 8.1260 s  5.8472 s < τ < 8.2215 s  6.2332 s < τ < 8.2936 s  6.0960 s < τ < 7.7680 s  6.5580 s < τ < 8.6920 s  6.3714 s < τ < 7.7312 s | τ > 8.2008 s  τ > 7.8176 s  τ > 7.8952 s  τ > 8.4772 s  τ > 8.1260 s  τ > 8.2215 s  τ > 8.2936 s  τ > 7.7680 s  τ > 8.6920 s  τ > 7.7312 s |
