# Supplementary material for: Ocular diagnostics and occipital neurovascular coupling in ocular hypertension and open angle glaucoma
Source: Front Neurosci. 2025 Dec 12;19:1689655. doi: 10.3389/fnins.2025.1689655 (PMC12740935; doi:10.3389/fnins.2025.1689655)
Supplement: Supplementary file 8 [file Table_2.docx]

**Supplementary Table ST2: summary of additional clinical characteristics (OCT variables).**

These characteristics were summarized using median, first and third quartiles (25-th and 75-th centiles, respectively). NORM=normal; OHT=ocular hypertensive; NTG=normal tension glaucoma; OAG=open angle glaucoma.

|  | **EYE** | **PATHOLOGICAL CLASSIFICATION** | | | |
| --- | --- | --- | --- | --- | --- |
|  |  | **NORM**  **(n=67)** | **OHT**  **(n=44)** | **NTG**  **(n=54)** | **OAG**  **(n=38)** |
| Macular OCT (µm)  Lower  Upper  Total | L  R  L  R  L  R | 31.0 ( 28.5 - 32.0)  31.0 ( 28.0 - 32.2)  30.0 ( 28.5 - 32.5)  30.0 ( 28.0 - 32.2)  30.0 ( 29.0 - 32.5)  30.5 ( 28.7 - 33.0) | 31.0 ( 29.0 - 33.0)  31.0 ( 29.5 - 32.0)  32.0 ( 29.0 - 33.0)  31.0 ( 29.0 - 33.0)  31.0 ( 29.0 - 33.0)  31.0 ( 29.0 - 32.0) | 23.0 (21.2 - 27.8)  25.0 (22.2 - 27.0)  25.0 (21.2 - 27.0)  27.0 (24.0 - 27.0)  24.0 (21.0 - 27.0)  25.5 (24.0 - 27.0) | 21.5 (20.8 - 29.2)  25.0 (20.0 - 29.0)  26.0 (20.0 - 29.0)  24.0 (22.0 - 29.0)  23.5 (20.0 - 29.0)  25.0 (21.0 - 28.0) |
| Peri-papillary OCT (µm)  Upper  Nasal  Lower  Temporal | L  R  L  R  L  R  L  R | 114.0 ( 98.0 -125.2)  113.0 (103.9 -122.5)  76.0 ( 61.0 - 85.0)  79.5 ( 68.8 - 84.0)  117.0 (106.5 -130.5)  122.0 (108.0 -131.9)  63.0 ( 58.0 - 67.5)  67.0 ( 62.8 - 72.0) | 115.0 (102.0 -122.0)  110.0 (103.0 -124.0)  72.0 ( 67.0 - 79.0)  67.0 ( 64.0 - 78.5)  118.0 (107.0 -130.0)  112.0 (104.0 -125.0)  66.0 ( 58.0 - 72.0)  65.0 ( 59.5 - 68.0) | 78.0 (58.5 - 95.5)  82.0 (70.5 - 92.0)  59.0 (44,0 - 66.5)  60.0 (55.5 - 64.5)  75.0 (63.0 - 90.2)  68.0 (63.0 - 77.0)  50.0 (41.5 - 62.0)  56.5 (47.8 - 63.0) | 72.0 (49.4 - 98.2)  70.5 (54.2 - 96.0)  56.0 (46.5 - 64.2)  51.5 (41.0 - 61.0)  73.5 (52.1 -102.5)  62.8 (48.2 - 93.8)  53.0 (34.0 - 64.8)  52.0 (42.2 - 72.8) |
