# Supplementary material for: Ocular diagnostics and occipital neurovascular coupling in ocular hypertension and open angle glaucoma
Source: Front Neurosci. 2025 Dec 12;19:1689655. doi: 10.3389/fnins.2025.1689655 (PMC12740935; doi:10.3389/fnins.2025.1689655)
Supplement: Supplementary file 9 [file Table_3.docx]

**Supplementary Table ST3.**

Median and quartiles (Q_1_, Q_3_) of TD-fNIRS parameters, according to signal type (O_2_Hb, HHb), acquisition channel (left/right hemisphere), and repetition of stimulation (1 to 5). NORM=normal; OHT=ocular hypertensive; NTG=normal tension glaucoma; OAG=open angle glaucoma

|  | **NORM** | | **OHT** | |
| --- | --- | --- | --- | --- |
|  | **LEFT EYE** | **RIGHT EYE** | **LEFT EYE** | **RIGHT EYE** |
|  | **τ_HHb_ [s]** | | | |
| **LEFT HEMISPHERE**  stimulation 1  stimulation 2  stimulation 3  stimulation 4  stimulation 5 | 3.2 (2.1, 6.4)  4.9 (2.6, 7.1)  6.1 (3.6, 7.8)  5.5 (3.0, 8.4)  4.8 (2.0, 7.0) | 5.4 (3.3, 7.1)  5.3 (4.0, 6.9)  6.1 (4.5, 7.3)  6.1 (3.4, 7.2)  5.8 (3.4, 7.5) | 4.3 (3.6, 6.7)  5.3 (3.6, 6.8)  5.4 (3.3, 6.5)  4.9 (1.8, 6.6)  5.5 (4.2, 7.6) | 4.3 (1.2, 6.5)  6.3 (3.6, 7.1)  6.1 (4.7, 8.0)  5.5 (3.7, 7.7)  5.2 (3.6, 6.8) |
| **RIGHT HEMISPHERE**  stimulation 1  stimulation 2  stimulation 3  stimulation 4  stimulation 5 | 4.1 (1.8, 6.3)  5.7 (0.8, 7.9)  5.5 (2.8, 6.8)  5.9 (3.7, 8.5)  5.4 (3.6, 7.1) | 5.5 (2.1, 8.3)  5.2 (3.3, 6.6)  6.3 (4.0, 7.4)  6.6 (4.9, 8.4)  6.2 (4.4, 7.3) | 2.8 (0.0, 5.5)  5.9 (3.9, 7.4)  4.5 (4.2, 6.0)  4.6 (1.6, 7.2)  5.3 (1.8, 7.2) | 6.0 (1.8, 7.3)  6.2 (1.2, 8.1)  5.3 (4.2, 7.3)  5.3 (4.3, 6.6)  5.7 (3.7, 8.0) |
|  | **τ_O2Hb_ [s]** | | | |
| **LEFT HEMISPHERE**  stimulation 1  stimulation 2  stimulation 3  stimulation 4  stimulation 5 | 3.8 (2.2, 6.1)  4.7 (3.4, 6.2)  4.4 (2.2, 7.0)  4.9 (3.1, 6.9)  5.9 (2.9, 7.2) | 4.4 (2.6, 6.0)  4.4 (3.4, 6.5)  5.2 (3.9, 6.2)  5.7 (3.9, 6.6)  5.0 (3.7, 6.7) | 4.0 (2.6, 5.8)  4.8 (3.5, 5.5)  3.4 (1.2, 5.8)  5.1 (3.7, 6.7)  4.8 (3.5, 6.4) | 3.5 (2.1, 5.2)  4.5 (1.8, 6.0)  5.0 (3.1, 6.6)  4.3 (2.6, 6.1)  5.2 (3.2, 6.9) |
| **RIGHT HEMISPHERE**  stimulation 1  stimulation 2  stimulation 3  stimulation 4  stimulation 5 | 4.6 (2.8, 5.9)  4.8 (3.5, 6.7)  4.3 (3.4, 6.1)  5.9 (4.1, 7.2)  4.5 (3.0, 6.2) | 5.4 (3.3, 7.8)  4.7 (3.6, 5.9)  5.3 (3.5, 6.8)  5.3 (3.8, 6.6)  5.3 (4.1, 6.6) | 4.0 (2.0, 5.5)  5.8 (4.7, 7.1)  4.9 (2.5, 6.3)  5.1 (2.7, 7.3)  5.5 (4.7, 6.5) | 4.7 (1.1, 5.8)  5.4 (2.4, 7.0)  4.0 (3.0, 6.3)  4.9 (3.2, 6.1)  4.5 (2.8, 6.4) |
|  | **A_HHb_ [**µ**M]** | | | |
| **LEFT HEMISPHERE**  stimulation 1  stimulation 2  stimulation 3  stimulation 4  stimulation 5 | -0.18 (-0.30, 0.00)  -0.24 (-0.37, -0.13)  -0.31 (-0.42, -0.18)  -0.17 (-0.42, -0.08)  -0.20 (-0.34, -0.10) | -0.18 (-0.30, -0.09)  -0.20 (-0.37, -0.14)  -0.30 (-0.44, -0.17)  -0.18 (-0.34, -0.09)  -0.23 (-0.38, -0.11) | -0.21 (-0.43, -0.11)  -0.24 (-0.38, -0.08)  -0.22 (-0.35, -0.05)  -0.18 (-0.34, -0.13)  -0.27 (-0.39, -0.09) | -0.16 (-0.31, -0.07)  -0.20 (-0.40, -0.05)  -0.26 (-0.33, -0.10)  -0.18 (-0.36, -0.08)  -0.12 (-0.40, 0.00) |
|  |  |  |  |  |
| **RIGHT HEMISPHERE**  stimulation 1  stimulation 2  stimulation 3  stimulation 4  stimulation 5 | -0.15 (-0.30, -0.01)  -0.16 (-0.36, 0.04)  -0.25 (-0.33, -0.16)  -0.23 (-0.45, -0.12)  -0.18 (-0.51, -0.09) | -0.16 (-0.35, -0.03)  -0.20 (-0.34, -0.13)  -0.23 (-0.57, -0.17)  -0.32 (-0.48, -0.15)  -0.31 (-0.45, -0.16) | -0.13 (-0.26, -0.06)  -0.24 (-0.33, -0.16)  -0.22 (-0.26, -0.03)  -0.19 (-0.38, -0.05)  -0.20 (-0.24, -0.09) | -0.19 (-0.31, -0.09)  -0.19 (-0.35, -0.07)  -0.21 (-0.36, -0.12)  -0.22 (-0.32, 0.01)  -0.20 (-0.33, -0.03) |
|  | **A_O2Hb_ [**µ**M]** | | | |
| **LEFT HEMISPHERE**  stimulation 1  stimulation 2  stimulation 3  stimulation 4  stimulation 5 | 0.49 (0.27, 0.92)  0.50 (0.30, 0.97)  0.66 (0.49, 1.09)  0.47 (0.36, 1.20)  0.73 (0.27, 1.04) | 0.59 (0.31, 0.93)  0.66 (0.28, 0.85)  0.69 (0.37, 1.10)  0.70 (0.39, 0.94)  0.67 (0.33, 1.04) | 0.56 (0.42, 0.91)  0.64 (0.43, 0.85)  0.58 (0.28, 0.88)  0.60 (0.36, 0.78)  0.53 (0.20, 0.89) | 0.56 (0.30, 1.04)  0.62 (0.15, 0.87)  0.61 (0.39, 0.83)  0.58 (0.24, 0.86)  0.56 (0.29, 0.97) |
| **RIGHT HEMISPHERE**  stimulation 1  stimulation 2  stimulation 3  stimulation 4  stimulation 5 | 0.64 (0.45, 0.95)  0.59 (0.31, 1.41)  0.64 (0.40, 0.94)  0.72 (0.38, 1.39)  0.75 (0.41, 1.24) | 0.68 (0.39, 1.26)  0.63 (0.34, 0.98)  0.72 (0.40, 1.26)  0.76 (0.39, 1.08)  0.83 (0.49, 1.21) | 0.69 (0.42, 1.26)  0.62 (0.43, 1.04)  0.50 (0.24, 0.93)  0.69 (0.26, 1.05)  0.72 (0.49, 0.89) | 0.66 (0.42, 1.05)  0.70 (0.52, 1.01)  0.56 (0.41, 0.86)  0.84 (0.27, 1.04)  0.52 (0.26, 0.91) |

|  | **NTG** | | **OAG** | |
| --- | --- | --- | --- | --- |
|  | **LEFT EYE** | **RIGHT EYE** | **LEFT EYE** | **RIGHT EYE** |
|  | τ**_HHb_ [s]** | | | |
| **LEFT HEMISPHERE**  stimulation 1  stimulation 2  stimulation 3  stimulation 4  stimulation 5 | 4.9 (1.5, 7.1)  4.2 (1.3, 9.0)  4.7 (2.1, 7.3)  5.1 (2.2, 8.2)  4.6 (1.7, 7.3) | 5.6 (1.4, 9.2)  4.5 (1.4, 7.7)  5.2 (1.7, 7.2)  4.7 (3.6, 7.1)  4.1 (2.8, 6.1) | 4.6 (1.1, 9.5)  4.0 (1.4, 6.6)  4.5 (1.2, 6.8)  6.3 (2.5, 9.3)  5.9 (3.2, 9.8) | 3.8 (2.2, 6.2)  4.8 (1.7, 6.6)  4.9 (1.9, 7.9)  3.4 (1.5, 7.1)  4.0 (0.8, 5.6) |
| **RIGHT HEMISPHERE**  stimulation 1  stimulation 2  stimulation 3  stimulation 4  stimulation 5 | 7.0 (2.6, 11.2)  5.3 (3.4, 7.8)  4.7 (2.9, 6.9)  4.2 (0.3, 7.4)  6.2 (1.6, 7.0) | 4.9 (3.4, 9.7)  4.9 (2.4, 6.5)  5.1 (1.7, 6.7)  6.3 (3.4, 7.9)  6.3 (4.4, 7.0) | 5.6 (1.6, 6.9)  6.0 (1.4, 7.7)  2.7 (1.9, 6.7)  5.7 (0.0, 7.8)  2.5 (0.4, 7.9) | 2.8 (2.3, 4.6)  6.7 (0.6, 8.6)  6.2 (2.5, 8.8)  6.7 (2.2, 8.7)  4.1 (0.6, 5.1) |
|  | τ**_O2Hb_ [s]** | | | |
| **LEFT HEMISPHERE**  stimulation 1  stimulation 2  stimulation 3  stimulation 4  stimulation 5 | 3.8 (2.6, 7.0)  7.0(3.4, 8.9)  3.0 (1.8, 6.5)  1.9 (0.1, 4.3)  4.9 (1.5, 6.5) | 4.6 (1.0, 7.3)  3.5 (1.1, 5.4)  4.2 (1.6, 6.5)  3.5 (2.3, 5.9)  5.1 (3.5, 7.9) | 4.5 (0.1, 6.1)  2.6 (0.7, 5.0)  3.9 (0.5, 8.5)  2.3 (0.0, 7.3)  6.2 (3.5, 8.5) | 3.2 (1.2, 5.8)  2.6 (1.9, 6.2)  5.7 (1.3, 8.8)  7.1 (4.0, 10.3)  5.5 (3.8, 7.5) |
| **RIGHT HEMISPHERE**  stimulation 1  stimulation 2  stimulation 3  stimulation 4  stimulation 5 | 6.0 (3.5, 8.6)  5.9 (3.3, 8.2)  3.7 (1.6, 5.2)  2.6 (0.4, 4.5)  4.5 (3.1, 6.4) | 3.8 (2.6, 6.8)  4.9 (3.3, 6.4)  3.3 (1.0, 5.3)  5.3 (2.9, 8.0)  5.7 (4.0, 7.0) | 4.9 (1.7, 6.0)  5.0 (2.7, 7.2)  4.5 (2.8, 6.5)  3.4 (1.2, 6.3)  4.0 (1.8, 7.4) | 2.6 (0.0, 5.2)  5.9 (2.0, 8.6)  5.7 (0.9, 7.4)  3.5 (1.2, 6.3)  4.3 (3.1, 7.2) |
|  | **A_HHb_ [**µ**M]** | | | |
| **LEFT HEMISPHERE**  stimulation 1  stimulation 2  stimulation 3  stimulation 4  stimulation 5 | -0.15 (-0.26, 0.00)  -0.11 (-0.18, 0.06)  -0.14 (-0.23, -0.05)  -0.11 (-0.15, 0.05)  -0.14 (-0.24, -0.03) | -0.09 (-0.18, -0.02)  -0.14 (-0.21, -0.04)  -0.11 (-0.21, -0.08)  -0.13 (-0.28, -0.06)  -0.14 (-0.31, -0.03) | -0.06 (-0.15, 0.03)  -0.06 (-0.21, 0.05)  -0.14 (-0.24, -0.03)  -0.04 (-0.19, 0.04)  -0.08 (-0.14, 0.08) | -0.05 (-0.11, 0.09)  -0.15 (-0.20, -0.09)  -0.11 (-0.18, 0.12)  -0.03 (-0.13, 0.06)  -0.09 (-0.25, 0.01) |
| **RIGHT HEMISPHERE**  stimulation 1  stimulation 2  stimulation 3  stimulation 4  stimulation 5 | -0.11 (-0.28, -0.02)  -0.14 (-0.20, -0.05)  -0.17 (-0.29, -0.06)  -0.09 (-0.25, -0.05)  -0.17 (-0.31, -0.04) | -0.13 (-0.22, -0.05)  -0.12 (-0.27, -0.03)  -0.17 (-0.23, -0.08)  -0.23 (-0.31, -0.11)  -0.19 (-0.36, -0.16) | -0.11 (-0.19, -0.08)  -0.08 (-0.17, -0.05)  -0.09 (-0.21, 0.02)  -0.05 (-0.19, 0.01)  -0.06 (-0.23, 0.05) | -0.10 (-0.16, -0.06)  -0.10 (-0.21, 0.04)  -0.07 (-0.13, 0.13)  -0.07 (-0.17, 0.09)  -0.13 (-0.28, -0.11) |
|  | **A_O2Hb_ [**µ**M]** | | | |
| **LEFT HEMISPHERE**  stimulation 1  stimulation 2  stimulation 3  stimulation 4  stimulation 5 | 0.26 (-0.16, 0.46)  0.26 ( 0.18, 0.77)  0.29 ( 0.16, 0.59)  0.16 (-0.04, 0.45)  0.48 ( 0.20, 0.65) | 0.46 (0.16, 0.62)  0.35 (0.16, 0.70)  0.30 (0.15, 0.64)  0.45 (0.13, 0.70)  0.57 (0.15, 0.78) | 0.22 (-0.13, 0.45)  0.17 (-0.01, 0.44)  0.18 ( 0.03, 0.42)  0.06 (-0.12, 0.40)  0.31 ( 0.11, 0.52) | 0.22 ( 0.17, 0.45)  0.25 ( 0.13, 0.41)  0.35 (-0.10, 0.54)  0.36 ( 0.09, 0.63)  0.34 (-0.04, 0.60) |
| **RIGHT HEMISPHERE**  stimulation 1  stimulation 2  stimulation 3  stimulation 4  stimulation 5 | 0.23 (-0.17, 0.57)  0.44 ( 0.22, 0.79)  0.40 ( 0.19, 0.64)  0.36 ( 0.17, 0.58)  0.38 ( 0.14, 0.76) | 0.37 (0.18, 0.69)  0.46 (0.08, 0.70)  0.37 (0.09, 0.58)  0.50 (0.22, 0.65)  0.63 (0.39, 0.87) | 0.25 (-0.01, 0.37)  0.17 ( 0.07, 0.52)  0.32 ( 0.20, 0.41)  0.22 ( 0.04, 0.30)  0.23 (-0.14, 0.54) | 0.25 (0.09, 0.48)  0.21 (0.15, 0.50)  0.23 (0.08, 0.69)  0.32 (0.15, 0.49)  0.38 (0.25, 0.57) |
