# Supplementary material for: Ocular diagnostics and occipital neurovascular coupling in ocular hypertension and open angle glaucoma
Source: Front Neurosci. 2025 Dec 12;19:1689655. doi: 10.3389/fnins.2025.1689655 (PMC12740935; doi:10.3389/fnins.2025.1689655)
Supplement: Supplementary file 10 [file Table_4.docx]

**Supplementary Table ST4: distribution of cluster membership within each terminal node**

Terminal nodes are shown in Supplementary Fig. 3. The numbers in the table are the number of eyes belonging to each cluster/terminal node couple. The percentages are classification rates, that is, the proportions of eyes belonging to the various clusters over the total number of eyes within each terminal node. HRF = hemodynamic response function.

| **CLUSTER** | **TERMINAL NODE** | | | | | | | |
| --- | --- | --- | --- | --- | --- | --- | --- | --- |
|  | **1** | **2** | **3** | **4** | **5** | **6** | **7** | **8** |
| **1: lowest neurovascular coupling** | 27 (84.4%) | 1 ( 8.3%) | 3 ( 9.4%) | 3 ( 6.4%) | 0 (0.0%) | 1 (11.1%) | 0 ( 0.0%) | 0 ( 0.0%) |
| **2: low neurovascular coupling** | 3 ( 9.4%) | 5 (41.7%) | 18 (56.2%) | 12 (25.5%) | 0 (0.0%) | 1 (11.1%) | 0 ( 0.0%) | 1 ( 3.1%) |
| **3: intermediate neurovascular coupling** | 1 ( 3.1%) | 1 ( 8.3%) | 3 ( 9.4%) | 29 (61.7%) | 11 (91.7%) | 0 (0.0%) | 4 (14.8%) | 1 ( 3.1%) |
| **5: high neurovascular coupling** | 0 ( 0.0%) | 0 ( 0.0%) | 3 ( 9.4%) | 3 ( 6.4%) | 0 (0.0%) | 4 (44.4%) | 19 (70.4%) | 5 (15.6%) |
| **6: highest neurovascular coupling** | 0 ( 0.0%) | 0 ( 0.0%) | 1 ( 3.1%) | 0 ( 0.0%) | 1 (8.3%) | 0 (0.0%) | 3 (11.1%) | 21 (65.6%) |
| **4: incoherent**  **HRF** | 1 ( 2.1%) | 5 (41.7%) | 4 (12.5%) | 0 ( 0.0%) | 0 (0.0%) | 3 (33.3%) | 1 ( 3.7%) | 4 (12.5%) |
| **Sum** | 32 (100%) | 12 (100%) | 32 (100%) | 47 (100%) | 12 (100%) | 9 (100%) | 27 (100%) | 32 (100%) |
