# Supplementary material for: Ocular diagnostics and occipital neurovascular coupling in ocular hypertension and open angle glaucoma
Source: Front Neurosci. 2025 Dec 12;19:1689655. doi: 10.3389/fnins.2025.1689655 (PMC12740935; doi:10.3389/fnins.2025.1689655)
Supplement: Supplementary file 11 [file Table_5.docx]

**SUPPLEMENTARY TABLE ST5: distribution of clusters within clinical classification and GSS2 perimetric classification**

The number and respective percentage of eyes belonging to the six clusters are reported, within each diagnostic group and within each stage.

HRF = hemodynamic response function. ; NORM=normal; OHT=ocular hypertensive; NTG=normal tension glaucoma; OAG=open angle glaucoma.

|  | **CLUSTER** | | | | | |  |
| --- | --- | --- | --- | --- | --- | --- | --- |
|  | **1: lowest**  **neurovascular**  **coupling** | **2:  low**  **neurovascular**  **coupling** | **3: intermediate neurovascular coupling** | **5: high**  **neurovascular**  **coupling** | **6: highest**  **neurovascular**  **coupling** | **4: incoherent**  **HRF** | **TOTAL** |
| **diagnosis**  NORM  OHT  NTG  OAG  oooled | 4 ( 6.0%)  5 (11.4%)  11 (20.4%)  15 (39.5%)  **35 (17.2%)** | 9 (13.4%)  5 (11.4%)  12 (22.2%)  14 (36.8%)  **40 (19.7%)** | 15 (22.4%)  13 (29.5%)  18 (33.3%)  4 (10.5%)  **50 (24.6%)** | 19 (28.4%)  11 (25.0%)  3 ( 5.6%)  1 ( 2.6%)  **34 (16.7%)** | 15 (22.4%)  6 (13.6%)  4 ( 7.4%)  0 ( 0.0%)  **25 (12.3%)** | 5 ( 7.5%)  4 ( 9.1%)  6 (11.1%)  4 (10.5%)  **19 (9.4%)** | **67 (100%)**  **44 (100%)**  **54 (100%)**  **38 (100%)**  **203** |
| **stage**  0  1  2  3  4  5  pooled | 7 (12.3%)  3 ( 5.9%)  9 (26.5%)  6 (18.8%)  5 (29.4%)  5 (41.7%)  **35 (17.2%)** | 7 (12.3%)  9 (17.6%)  9 (26.5%)  8 (25.0%)  6 (35.3%)  1 ( 8.3%)  **40 (19.7%)** | 11 (19.3%)  18 (35.3%)  6 (17.6%)  12 (37.5%)  1 ( 5.9%)  2 (16.7%)  **50 (24.6%)** | 17 (29.8%)  10 (19.6%)  5 (14.7%)  1 ( 3.1%)  1 ( 5.9%)  0 ( 0.0%)  **34 (16.7%)** | 11 (19.3%)  9 (17.6%)  2 ( 5.9%)  2 ( 6.2%)  0 ( 0.0%)  1 ( 8.3%)  **25 (12.3%)** | 4 ( 7.0%)  2 ( 3.9%)  3 ( 8.8%)  3 ( 9.4%)  4 (23.5%)  3 (25.0%)  **19 ( 9.4%)** | **57 (100%)**  **51 (100%)**  **34 (100%)**  **32 (100%)**  **17 (100%)**  **25 (100%)**  **203** |
