# Supplementary material for: Ocular diagnostics and occipital neurovascular coupling in ocular hypertension and open angle glaucoma
Source: Front Neurosci. 2025 Dec 12;19:1689655. doi: 10.3389/fnins.2025.1689655 (PMC12740935; doi:10.3389/fnins.2025.1689655)
Supplement: Supplementary file 12 [file Table_6.docx]

**Supplementary Table ST6: characterization of clusters according to clinical examination results**

IOP and PAC were summarized using mean and standard deviation, and the remainder variables were summarized by median and quartiles. M_OCT = macular OCT; P_OCT = peri-papillary OCT; lVEPs = VEPs latency; aVEPs = VEPs amplitude; lPERG = PERG latency; aPERG = PERG amplitude, HRF = hemodynamic response function.

|  | **CLUSTER** | | | | | |
| --- | --- | --- | --- | --- | --- | --- |
|  | **1: lowest neurovascular**  **coupling** | **2: low**  **neurovascular coupling** | **3: intermediate neurovascular coupling** | **5:  high**  **neurovascular coupling** | **6: highest neurovascular coupling** | **4: incoherent**  **HRF** |
| IOP (mm) | 14.3, 2.7 | 14.8, 3.2 | 14.5, 3.0 | 15.6, 2.8 | 15.6, 3.5 | 13.8, 3.9 |
| PAC (μm) | 559.2, 37.7 | 552.4, 34.0 | 551.5, 38.4 | 558.7, 37.5 | 551.7, 43.4 | 547.9, 37.5 |
| MD (dB) | 6.9 ( 3.2 - 13.2) | 5.1 ( 3.3 - 9.0) | 3.6 ( 2.4 - 7.4) | 2.8 ( 1.7 - 3.8) | 3.2 ( 1.6 - 3.7) | 6.5 ( 4.0 - 15.0) |
| CLV (dB^2^) | 3.7 ( 1.9 - 6.5) | 3.7 ( 1.8 - 5.6) | 2.3 ( 1.8 - 4.7) | 1.6 ( 1.4 - 2.2) | 1.8 ( 1.4 - 2.3) | 4.4 ( 1.9 - 7.8) |
| M_OCT (μm)  Lower  Upper  Total  P_OCT (μm)  Upper  Nasal  Lower  Temporal | 27.0 ( 24.0 - 30.5)  27.0 ( 23.5 - 29.0)  27.0 ( 23.5 - 30.0)  82.0 ( 62.5 -112.5)  58.0 ( 41.5 - 67.0)  85.0 ( 63.0 -108.0)  62.0 ( 49.0 - 70.0) | 25.0 ( 21.0 - 30.0)  26.0 ( 22.0 - 29.0)  26.0 ( 21.0 - 29.0)  91.0 ( 77.6 -112.2)  63.0 ( 54.5 - 79.0)  81.5 ( 65.0 -118.4)  56.0 ( 43.2 - 65.2) | 30.0 ( 25.0 - 31.0)  29.0 ( 26.0 - 32.0)  29.0 ( 26.0 - 32.0)  103.8 ( 85.2 -117.6)  69.0 ( 60.2 - 76.8)  109.5 ( 76.2 -123.9)  61.0 ( 52.0 - 66.0) | 27.0 ( 24.0 - 30.5)  27.0 ( 23.5 - 29.0)  27.0 ( 23.5 - 30.0)  111.8 (102.0 -121.9)  76.0 ( 64.2 - 82.0)  114.5 (105.5 -126.8)  66.0 ( 60.2 - 69.0) | 25.0 ( 21.0 - 30.0)  26.0 ( 22.0 - 29.0)  26.0 ( 21.0 - 29.0)  105.0 (100.0 -115.0)  68.0 ( 64.0 - 80.0)  114.0 (102.0 -132.0)  63.0 ( 59.0 - 71.0) | 30.0 ( 25.0 - 31.0)  29.0 ( 26.0 - 32.0)  29.0 ( 26.0 - 32.0)  83.0 ( 48.5 -106.0)  54.0 ( 43.0 - 61.0)  73.0 ( 62.5 -110.8)  65.0 ( 51.0 - 68.5) |
| lVEP30 (ms) | 120.0 (113.0 -133.0) | 118.5 (110.8 -125.8) | 113.5 (109.0 -117.0) | 114.0 (107.2 -118.0) | 112.0 (105.0 -115.0) | 109.0 (107.0 -119.5) |
| lVEP15 (ms) | 125.0 (117.5 -138.5) | 120.0 (116.2 -128.5) | 119.5 (114.2 -125.0) | 118.0 (115.0 -120.8) | 118.0 (112.0 -120.0) | 119.0 (113.0 -121.0) |
| aVEP30 (μV) | 7.8 ( 5.2 - 9.9) | 7.1 ( 4.6 - 11.2) | 7.8 ( 5.4 - 9.9) | 8.4 ( 6.1 - 14.2) | 8.4 ( 6.8 - 11.8) | 6.5 ( 5.6 - 11.4) |
| aVEP15 (μV) | 7.2 ( 3.9 - 9.4) | 7.0 ( 4.6 - 11.3) | 8.9 ( 5.8 - 11.4) | 11.4 ( 7.3 - 15.1) | 9.4 ( 7.7 - 13.5) | 8.7 ( 5.7 - 11.8) |
| lPERG30 (ms) | 54.0 ( 50.5 - 61.5) | 58.5 ( 56.0 - 63.0) | 61.0 ( 57.0 - 65.0) | 58.0 ( 55.0 - 62.0) | 58.0 ( 54.0 - 61.0) | 61.0 ( 56.0 - 67.0) |
| lPERG15 (ms) | 63.0 ( 58.5 - 68.0) | 60.5 ( 55.0 - 68.5) | 62.0 ( 55.0 - 69.8) | 62.0 ( 57.5 - 67.8) | 62.0 ( 58.0 - 65.0) | 60.0 ( 55.5 - 68.0) |
| aPERG30 (μV) | 2.4 ( 1.9 - 3.2) | 2.4 ( 1.6 - 2.8) | 2.8 ( 1.9 - 3.4) | 2.8 ( 2.0 - 3.4) | 2.8 ( 2.2 - 3.5) | 2.5 ( 1.5 - 3.0) |
| aPERG15 (μV) | 1.9 ( 1.4 - 2.4) | 2.2 ( 1.7 - 2.7) | 2.3 ( 1.9 - 2.8) | 2.1 ( 1.8 - 2.8) | 1.9 ( 1.7 - 2.4) | 2.2 ( 1.6 - 2.5) |
